# Molecular subtypes of Alzheimer’s disease

**DOI:** 10.1038/s41598-018-21641-1

**Published:** 2018-02-19

**Authors:** Giuseppe Di Fede, Marcella Catania, Emanuela Maderna, Roberta Ghidoni, Luisa Benussi, Elisa Tonoli, Giorgio Giaccone, Fabio Moda, Anna Paterlini, Ilaria Campagnani, Stefano Sorrentino, Laura Colombo, Adriana Kubis, Edoardo Bistaffa, Bernardino Ghetti, Fabrizio Tagliavini

**Affiliations:** 1 IRCCS Foundation “Carlo Besta” Neurological Institute, Milan, Italy; 2 Molecular Markers Laboratory, IRCCS Istituto Centro San Giovanni di Dio - Fatebenefratelli, Brescia, Italy; 3 Department of Molecular Biochemistry and Pharmacology, IRCCS Istituto di Ricerche Farmacologiche “Mario Negri”, Milan, Italy; 4 Department of Toxicology, Wroclaw Medical University, Wrocław, Poland; 5 Department of Pathology and Laboratory Medicine, Indiana University, Indianapolis, Indiana, USA

## Abstract

Protein misfolding and aggregation is a central feature of several neurodegenerative disorders including Alzheimer’s disease (AD), in which assemblies of amyloid β (Aβ) peptides accumulate in the brain in the form of parenchymal and/or vascular amyloid. A widely accepted concept is that AD is characterized by distinct clinical and neuropathological phenotypes. Recent studies revealed that Aβ assemblies might have structural differences among AD brains and that such pleomorphic assemblies can correlate with distinct disease phenotypes. We found that in both sporadic and inherited forms of AD, amyloid aggregates differ in the biochemical composition of Aβ species. These differences affect the physicochemical properties of Aβ assemblies including aggregation kinetics, resistance to degradation by proteases and seeding ability. Aβ-amyloidosis can be induced and propagated in animal models by inoculation of brain extracts containing aggregated Aβ. We found that brain homogenates from AD patients with different molecular profiles of Aβ are able to induce distinct patterns of Aβ-amyloidosis when injected into mice. Overall these data suggest that the assembly of mixtures of Aβ peptides into different Aβ seeds leads to the formation of distinct subtypes of amyloid having distinctive physicochemical and biological properties which result in the generation of distinct AD molecular subgroups.

## Introduction

Misfolding, aggregation and deposition of amyloid β (Aβ) peptides in brain parenchyma and vessel walls are regarded as key events in the pathogenic cascade of Alzheimer’s disease (AD)^1,2^. Aβ fragments of various lengths are generated by cleavage of the amyloid precursor protein (APP) by β- and γ-secretases, and subsequent digestion by endogenous proteases. This process results in the formation of a variety of N- and C-terminal-truncated Aβ species^1,3–5^ having the ability to assemble into abnormal aggregates^6–8^.

During the past few years, a great number of studies have pointed to the Aβ aggregates as key determinants in the molecular machinery leading to AD^9,10^. Moreover, it has been suggested that different Aβ assemblies exist, each defined by distinct molecular size, stability and neurotoxic properties^11^. However, their specific relevance in AD pathogenesis is unclear. In addition, the existence of different N-terminal and C-terminal truncated forms of Aβ in AD brain is now well known^12,13^. As for Aβ aggregates, we actually don’t know if different Aβ monomeric isoforms play a role in determining specific molecular AD phenotypes.

AD comprises different phenotypes characterized by diverse clinical presentations, neuroanatomical involvement and neuropathological profiles. Although this phenotypic heterogeneity is most striking in the dominantly inherited forms, it is also well recognized in sporadic cases. The molecular basis of these phenotypic variations is largely unknown^14–16^.

Previous studies showed that Aβ deposits differ in morphology and biochemical composition among individuals with AD, and among APP transgenic mouse models^17–22^. The existence of different Aβ “morphotypes” is further supported by the finding of distinct structural variants of Aβ fibrils isolated from brain of AD patients^23,24^.

It has been hypothesized that spread of Aβ aggregates from region to region may account for propagation of the disease process and neurodegeneration, with mechanisms analogous to spreading of the pathogenic forms of the prion protein (PrP^Res^) in transmissible spongiform encephalopathies^25^. This hypothesis is based on experimental evidence that Aβ amyloidosis can be induced in animal models by inoculation of brain extracts containing aggregated Aβ^26,27^, and distinct types of Aβ aggregates can reproduce the neuropathological profile of the donor in a given transgenic mouse line^28^.

The aims of this study were: (i) to investigate whether a molecular heterogeneity based on the existence of distinct profiles of Aβ aggregates occurs in AD; (ii) to define the molecular features characterizing distinct Aβ assemblies and test the hypothesis that differences in molecular profiles affect the physicochemical properties of Aβ assemblies, which can be involved in the generation of different AD phenotypes.

We found that in both sporadic and genetically determined forms of AD, amyloid aggregates show differences in the biochemical composition of Aβ species. Such differences are associated with changes in aggregation kinetics, resistance to protease degradation, seeding activity *in vitro*, and ability to induce amyloidosis in animal models.

These findings support the hypothesis that the variability of AD phenotypes may result from a potential multiplicity of Aβ aggregation modalities. Accordingly, a detailed analysis of Aβ aggregation and seeding properties may lead to a novel classification of AD, based on the identification of subtypes of Aβ’s distinct macromolecular aggregates.

## Results

### AD patients are highly heterogeneous regarding the morphologic characteristics of Aβ deposits

**Figure 1:**
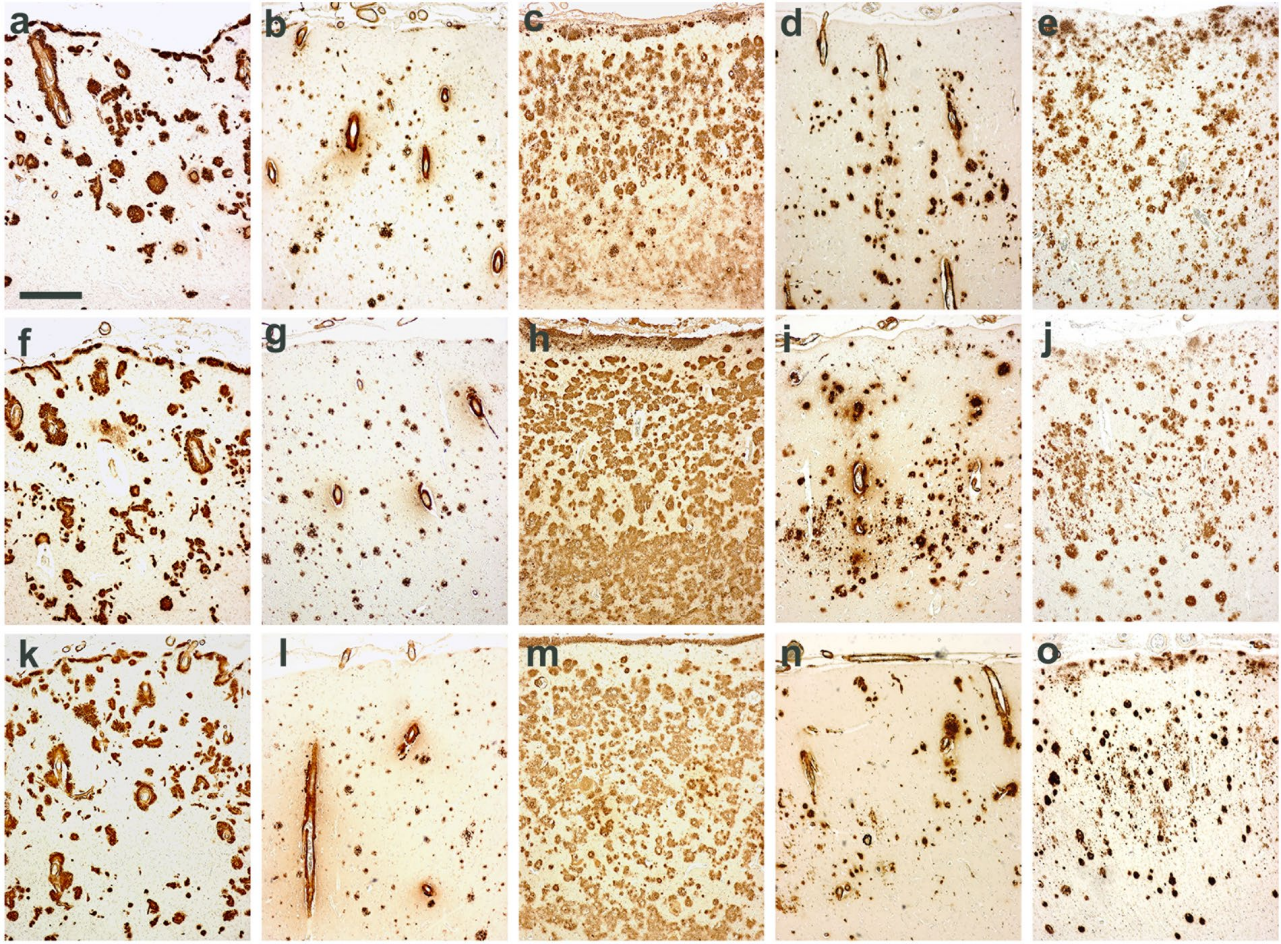
Differences in amyloid-related pathology of AD cases. (**a,f,k**) APP_A673V_ (fAD1 in Table S1); (**b,g,l**) APP_A713V_ (fAD2 in Table S1); (**c,h,m**) PS1_P117A_ (fAD3 in Table S1); (**d,i,n**) sAD carrying the ApoE ε4/ε4 genotype (sAD1 in Table S1); (**e,j,o**) sAD ε3/ε3 (sAD6 in Table S1). Scale bar = 400 um. (**a,b,c,d,e**) frontal cortex; (**f,g,h,i,j**) temporal cortex; (**k,l,m,n,o**) occipital cortex. Immunohistochemical study perfomed using the 4G8 antibody against Aβ.

A neuropathological study in a cohort consisting of 20 patients with sporadic AD (indicated as sAD1-sAD20 numbered in Table S1), and 4 patients with familial AD associated with APP, PS1 or PS2 mutations - fAD-APP_A673V_, fAD-APP_A713T_, fAD-PS1_P117A_ and fAD-PS2_A85V_ – (indicated as fAD1–4 in Table S1), showed the common typical changes of AD consisting of parenchymal (*amyloid plaques*) and vascular (*congophilic amyloid angiopathy*, *CAA*) amyloid deposits, neurofibrillary tangles, neuropil threads and dystrophic neurites containing hyperphosphorylated tau, accompanied by neuronal loss, astrogliosis and microglial activation throughout the cerebral cortex. However, an in-depth examination of the amyloid-β pathology revealed the existence of dissimilarities concerning density, shape and size as well as the relative severity of parenchymal versus vascular deposition of amyloid in the brain (Fig. 1). In particular, the A673V mutation (fAD1) (a,f,k panels), whose full neuropathological assessment was previously described^29^, showed abundant amyloid deposits both in the parenchyma and in the vessels, immunoreactive for antibodies recognizing epitopes spanning overall the Aβ sequence. Many small vessels in parenchyma and leptomeninges showed thickening of the walls due to the accumulation of amyloid and ‘drusige Entartung’. Pathological hallmarks of APP_A713T_ (fAD2) (b,g,l panels) were CAA and low-density parenchymal Aβ amyloid deposits in the neuropil. Severe amyloid deposition affected leptomeningeal and small parenchymal vessels in the cerebral hemisphere. Affected vessels were disrupted with amyloid assuming a radial appearance, thickening and double barreling of the wall, loss of smooth muscle cells and narrowing of the lumina. Neuropathological analysis of the PS1_P117A_ case (fAD3) (c,h,m panels) was remarkable for numerous and widespread plaques in all cortical layers with higher density in the subpial region. The two sporadic cases showed completely different amyloid patterns, one (sAD1) (d,i,n panels) with predominance of vascular amyloid deposits and capillary Aβ deposition spreading from the vessel walls into the surrounding neuropil (‘drusige Entartung’), and mature plaques sparse in cerebral cortex, the other (sAD6) (e,j,o panels) characterized by tiny and diffuse plaques distributed over all cortical layers, with focal and mild CAA.

This observation suggested that, just considering the morphology and distribution of Aβ deposits, the neuropathology of AD is extremely variable not only among genetic cases but also among sporadic patients.

### AD patients have distinct amyloid-β profiles

Following the hypothesis that changes in Aβ pathology may be due to differences in the molecular composition of amyloid, we extracted and purified parenchymal amyloid from the brains of the sAD1–20 and the fAD1–4 patients and analyzed its Aβ content by an immunoproteomic assay. The study revealed that different Aβ isoforms, including N- and C-terminally truncated species, contribute to amyloid composition and that the relative amounts of such peptides can vary among AD brains.

**Figure 2:**
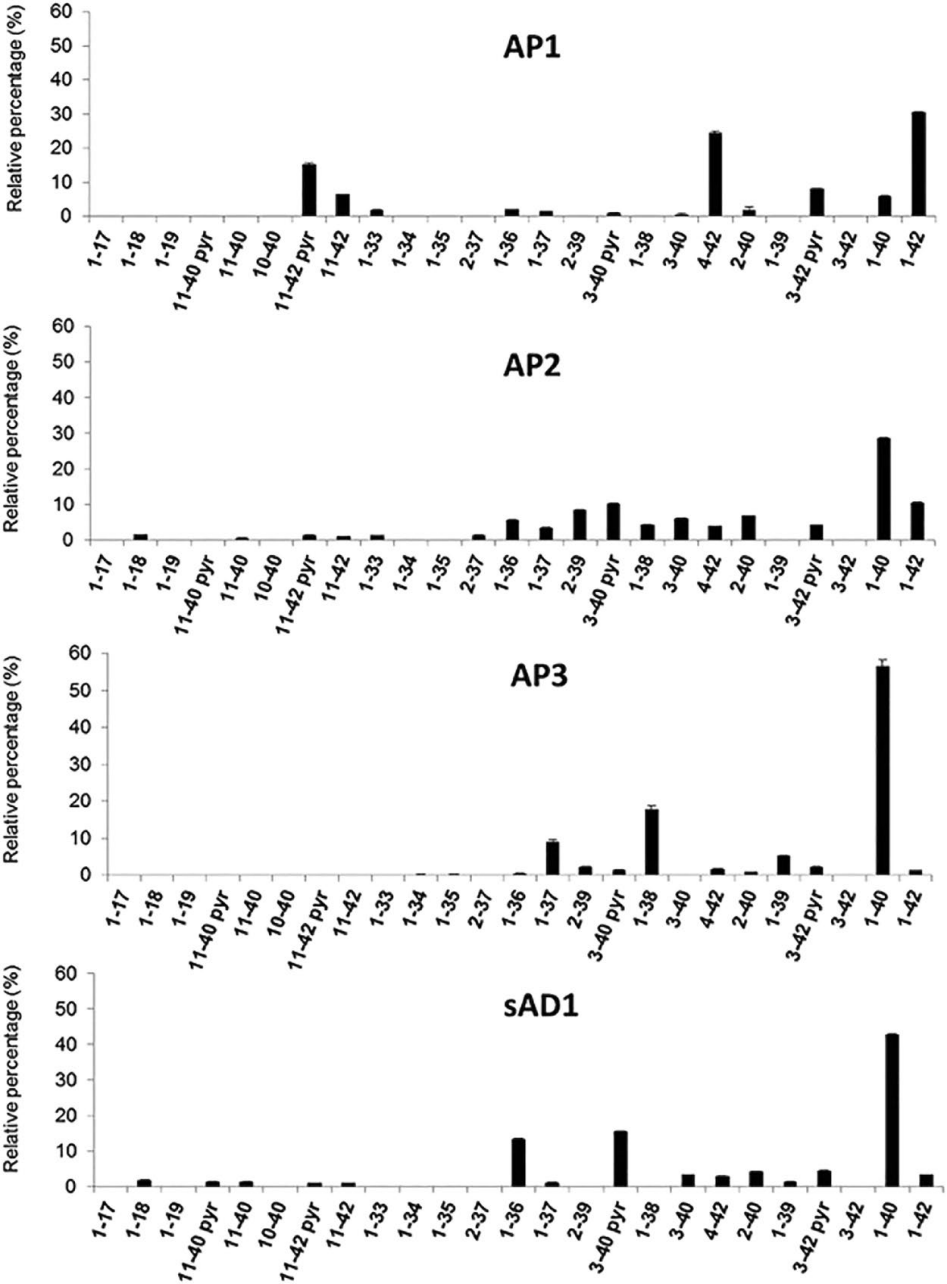
Aβ isoforms’ fingerprints. Aβ isoforms were analyzed by immunoproteomic analysis, performed using two different Aβ monoclonal antibodies (6E10 and 4G8) on pre-activated chip array, followed by mass spectrometry. Relative percentage of Aβ peptides (with respect to the total Aβ amount) as measured in human brains; for each AD subgroup a representative profile is reported (n = 3, mean relative percentages ± SEM (AP1 to AP3 numbering; sAD1 profile is reported outside of the other groups for its peculiarities).

These data led to the identification of two main AD subgroups, each characterized by distinctive profiles of Aβ species (Table 1 and Fig. 2), indicated as Amyloid Profile 1 (AP1) and Amyloid Profile 2 (AP2). AP1 was found in 14 sAD patients and in the individuals with presenilin mutations, while AP2 was detected in five sAD cases. AP1 was marked by a high relative proportion of AβX-42 peptides, especially Aβ1-42, Aβ4-42, Aβ11-42 and the pyroglutamate-modified Aβ3pE-42 and Aβ11pE-42, while AP2 was distinguished by the presence of both AβX-40 and AβX-42 peptides, with a prevalence of the former, and minor species including N- and C-terminal truncated forms, such as Aβ2-39. The two patients with the APP mutations showed a distinct amyloid profile, designated as AP3, mainly composed by Aβ1-40, Aβ1-38 and Aβ1-37. Finally, one individual with sAD (sAD1), neuropathologically characterized by very severe CAA (panels d,i,n in Fig. 1) exhibited a peculiar profile with a predominance of Aβ1-40, Aβ3pE-40 and Aβ1-36 isoforms.

**Table 1:** Molecular grouping of Alzheimer’s disease cases based on Aβ content in amyloid.

Profile	Case	Main Aβ peptides	*Apo E* genotype
AP1	fAD3 (*PS1*_P117A_), fAD4 (*PS2*_A85V_), sAD2, sAD3, sAD4, sAD5, sAD6, sAD7, sAD8, sAD10, sAD12, sAD14, sAD15, sAD18, sAD19, sAD20	Aβ1-42, Aβ4-42, Aβ3pE-42, Aβ11pE-42	ε2 = 9.4% ε3 = 68.7% ε4 = 21.9% ε2 = 10.0%
AP2	sAD9, sAD11, sAD13, sAD16, sAD17	Aβ1-40, Aβ1-42	ε3 = 30.0% ε4 = 60.0%
AP3	fAD1(*APP*_A673V_), fAD2 (*APP*_A713T_)	Aβ1-40, Aβ1–38, Aβ1-37	ε3 = 100%
—	sAD1	Aβ1-40, Aβ3pE-40, Aβ1-36	ε4 = 100%

According to these data, Table 1 shows a molecular stratification of our series of AD cases into three distinct subgroups (AP1-AP3). sAD1, whose molecular profile cannot be included in the other groups, is also reported.

As detailed in the “*Supplementary Information*” online section, neuropathological studies provided evidence of heterogeneity of patterns of Aβ deposition among both familial and sporadic AD patients (Table S1). However, when we searched for correlations between the molecular and the neuropathological profiles within the same AD subgroup, we did not find a clear-cut link between the two variables. These data suggest that the pathologic changes occurring in AD brains are the result of a combination of molecular mechanisms involving not only Aβ peptides but also additional molecules, such as tau and other unexplored or thoroughly unknown factors.

### Distinct molecular profiles of AD show differences in their aggregation pathways

**Figure 3:**
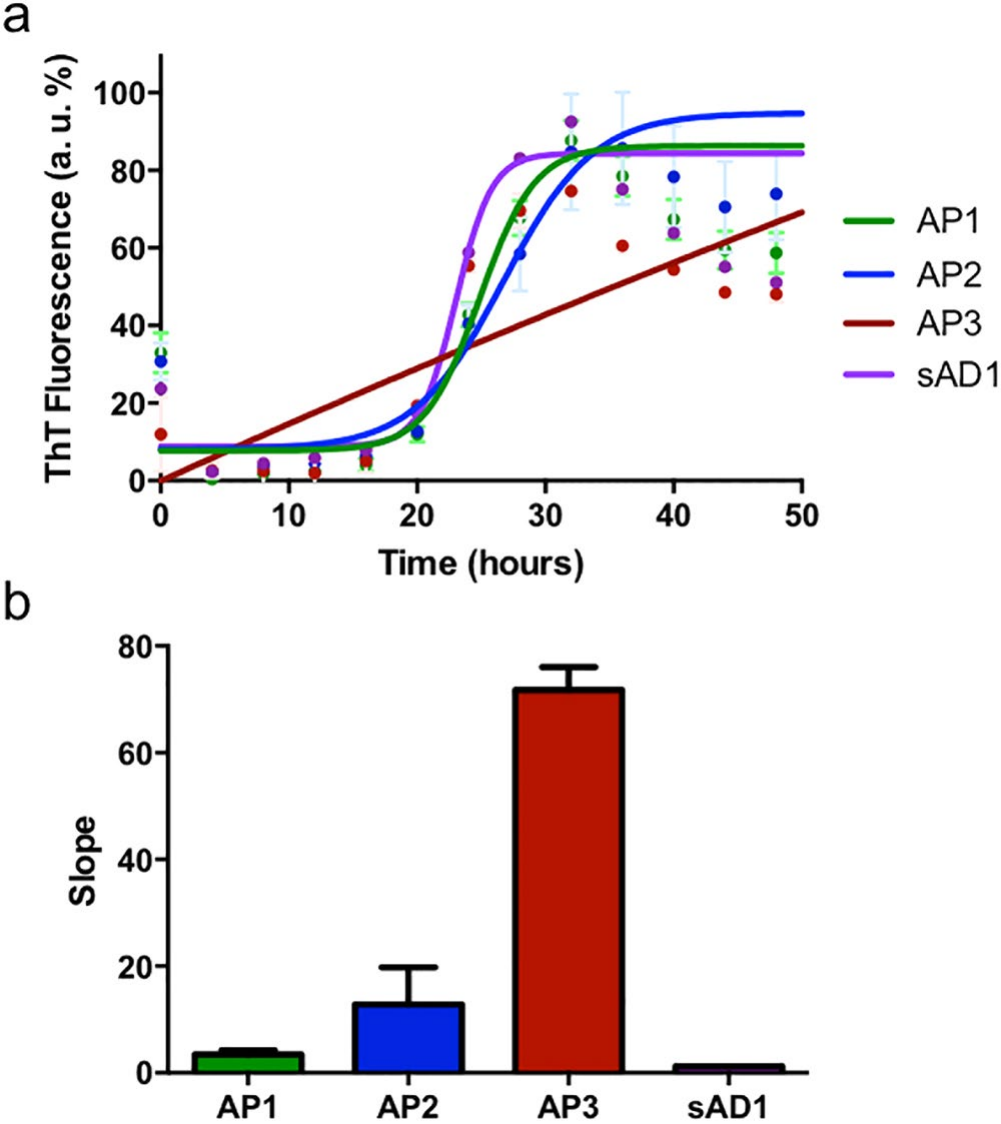
Aggregation pathways of different Aβ seeds by ThT assays. Aggregation kinetics of distinct AD Aβ profiles (AP1-AP3 and sAD1 case) were analyzed by ThT assays. Soluble fractions from AD brain homogenates were diluted in 100 mM Tris-HCl pH 7.5, 5 μM ThT. ThT intensity was normalized to the corresponding maximal ThT fluorescence and fitted using Boltzmann equation (**a**). The kinetics were compared by considering the slope of the normalized curves described by ThT fluorescence emission (**b**).

We then investigated whether the soluble fraction of brain homogenates from patients with different Aβ profiles has different ability to aggregate *in vitro* using ThT assays. The analysis showed that *de novo* amyloidogenesis differed among the two main molecular subgroups of AD, in that the AP1 aggregation kinetics was faster than AP2 samples. The samples from APP-mutated patients (AP3) showed an aggregation pattern slower than the other subgroups during the time-course of the study. On the opposite, aggregation kinetics was especially rapid for sAD1 brain sample (Fig. 3a,b).

Brain extracts immunodepleted from Aβ did not show any aggregation, indicating that Aβ peptides are the aggregating species in the setting of this assays (data not shown).

### Distinct molecular subgroups of AD have different seeding abilities

**Figure 4:**
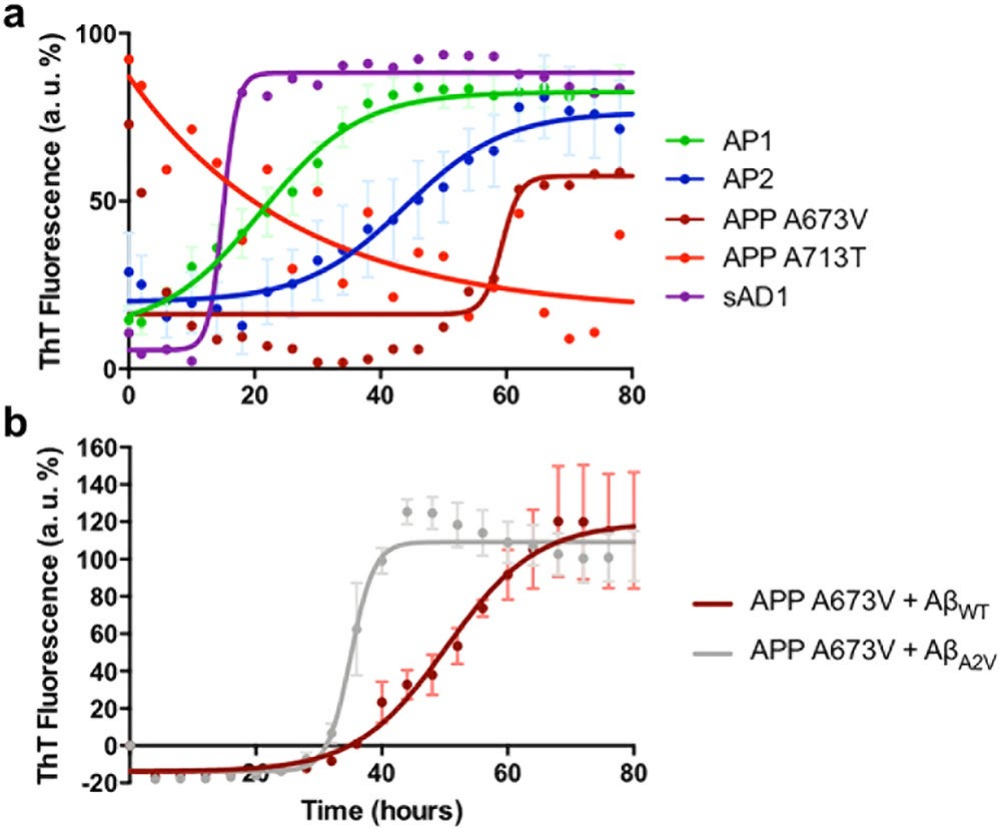
RT-QuIC profiles of human brain extracts from the molecular subgroups of AD. Soluble fractions from AD brain homogenates were diluted in 100 mM Tris-HCl pH 7.5, 5 μM ThT, 4 μM Aβ1-42_WT_ (panel a) or Aβ1-42_A2V_ (panel and **b**). ThT intensity was normalized to the corresponding maximal ThT fluorescence and expressed as relative arbitrary units (a. u. %). Comparison of aggregation kinetics of brain extracts from AP1 and AP2 profiles and APP_A673V_, APP_A713T_ and sAD1 subjects (**a**). Each brain sample was analyzed in quadruplicate. Data are shown as mean ± SEM. Comparison of aggregation kinetics of APP_A673V_ brain extract when co-incubated with Aβ1-42 wild-type or Aβ1-42 carrying the A2V mutation, corresponding to A673V substitution on APP gene (**b**). Each brain sample was analyzed in triplicate. Data are shown as mean ± SEM.

RT-QuIC was used to assess the seeding effects of the molecular subgroups of AD on synthetic wild-type Aβ1-42 (Aβ1-42_WT_) substrate (Fig. 4a). The study revealed that brain extracts from individuals belonging to AP1 and AP2, and the sAD1 case had seeding activities, since they shorten the lag phase of aggregation kinetics of synthetic Aβ1-42_WT_ (Fig. 4a). The results were mostly consistent with those observed in the previous experiment in that (i) AP1 showed a seeding activity higher than AP2 and (ii) the sAD1 sample induced the fastest aggregation of Aβ1-42 which, under this experimental setting, showed a steeper slope in the polymerization step of kinetics. In contrast, brain extracts from APP-mutated patients (AP3) showed a weak ability of inducing the aggregation of the Aβ1-42_WT_ substrate and followed different aggregation kinetics. Interestingly, the aggregation kinetics induced by the brain extract from the APP_A673V_ homozygous patient are faster when we used the mutated Aβ peptide (i.e., Aβ1-42 carrying the A673V mutation) as substrate in the RT-QuIC assay, suggesting that the affinity between seed and monomer is one of the modifiers of the aggregation profile (Fig. 4b).

APP_A713T_ shows high ThT signal at the beginning of the assay. This may be due to a very fast recruitment of the substrate that leads to the early saturation of ThT. The decrease of the signal in the rest of the curve may be caused by the instability of the aggregates generated by this brain extract along the course of the assay.

RT-QuIC performed on Aβ1-42_WT_ peptide co-incubated with brain extracts from control group (i.e., nondemented subjects without neuropathological changes) and from immunodepleted controls (Fig. S1) did not show any aggregation during the time course of the experiments.

RT-QuIC assay was also carried out using a synthetic wild-type Aβ1-40 substrate (Fig. S2). In this case, no substantial differences were observed among the brain extracts of the different subgroups, suggesting that Aβ1-40 is not a useful substrate to detect variances in seeding abilities of distinct AD subgroups.

### Aβ aggregates from AD molecular subgroups show different resistance to degradation

**Figure 5:**
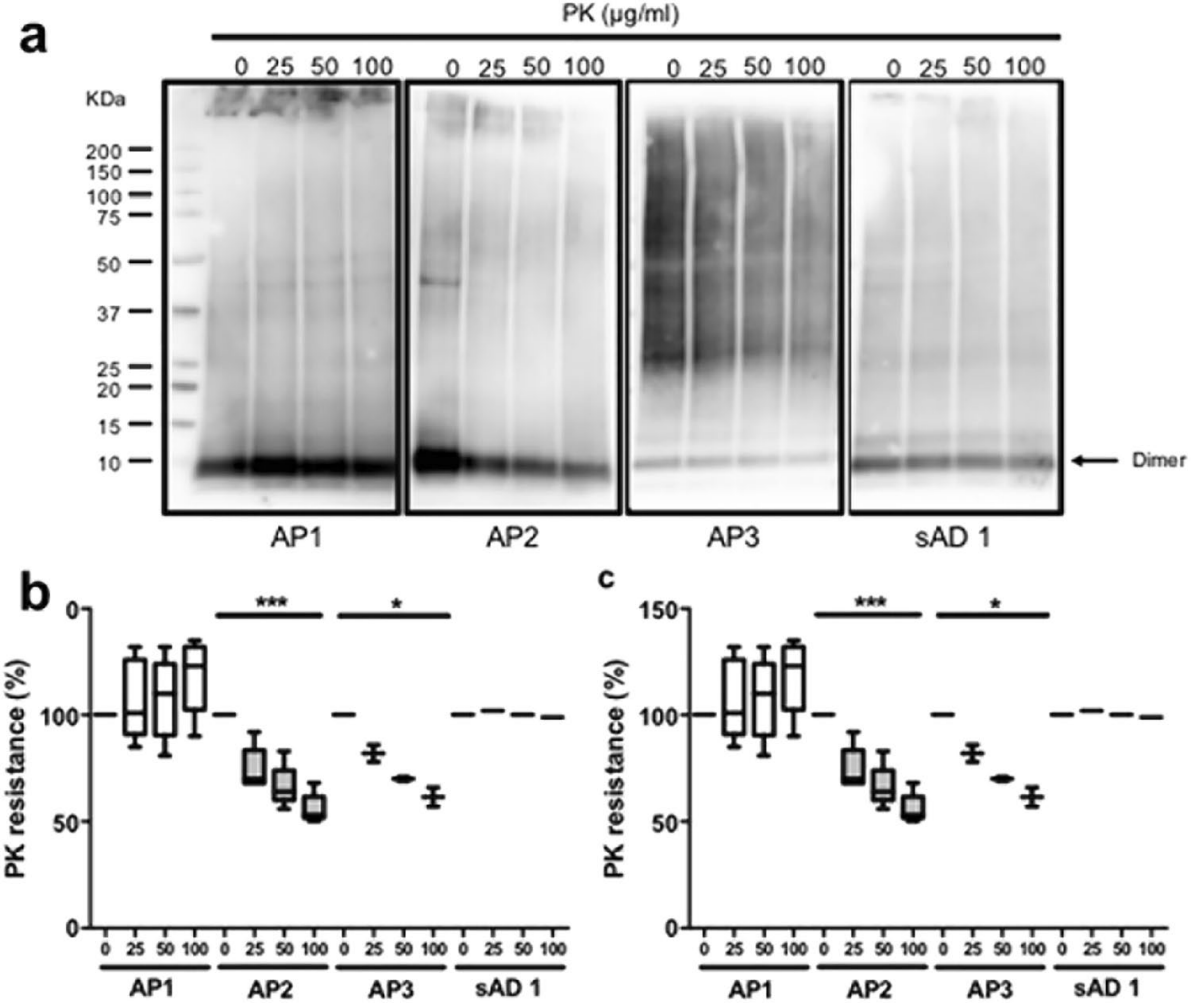
PK resistance of Aβ-containing human brain extracts. Insoluble fractions from AD brain homogenates were digested with 0, 25, 50, 100 µg/ml of PK and analyzed by Western blot using 4G8 antibody. The signal intensity of all the Aβ aggregates (**a**,**b**) or of Aβ dimers (**a**,**c**) was quantified by densitometry; data were compared by two-ways ANOVA (*p < 0.05; ***p < 0.001). Each blot shows the digestion of one brain extract representative of each amyloid profile. The blots were cropped; the original blots are shown in Supplementary Figure S3.

The resistance of Aβ aggregates to proteolysis was assessed by digestion of P3 fraction from AD brain homogenates with increasing amounts of PK. Samples belonging to AP1 subgroup and the sAD1 case showed very high resistance to degradation, since increasing doses of PK (up to 100 μg/ml) did not affect Aβ aggregates. Conversely, a clear dose-dependent PK degradation of Aβ assemblies was observed in samples from the AP2 subgroup and the two patients carrying APP mutations (Fig. 5a,b, S3).

A similar effect was found for the digestion of Aβ dimers (Fig. 5a,c), as AP1 subgroup and the sAD1 case were quite stable over increasing PK concentrations, AP2 underwent a dose-dependent proteolysis, and the dimers from the APP-mutated cases were partially degraded by PK.

These data indicate that differences in the Aβ composition of amyloid may affect the proteolytic activity of endogenous proteases on Aβ aggregates, making the Aβ assemblies derived from distinct molecular subgroups of AD more or less stable.

### Intracerebral injection of brain extracts from human subgroups in mice results in distinctive pattern of amyloid deposition

**Figure 6:**
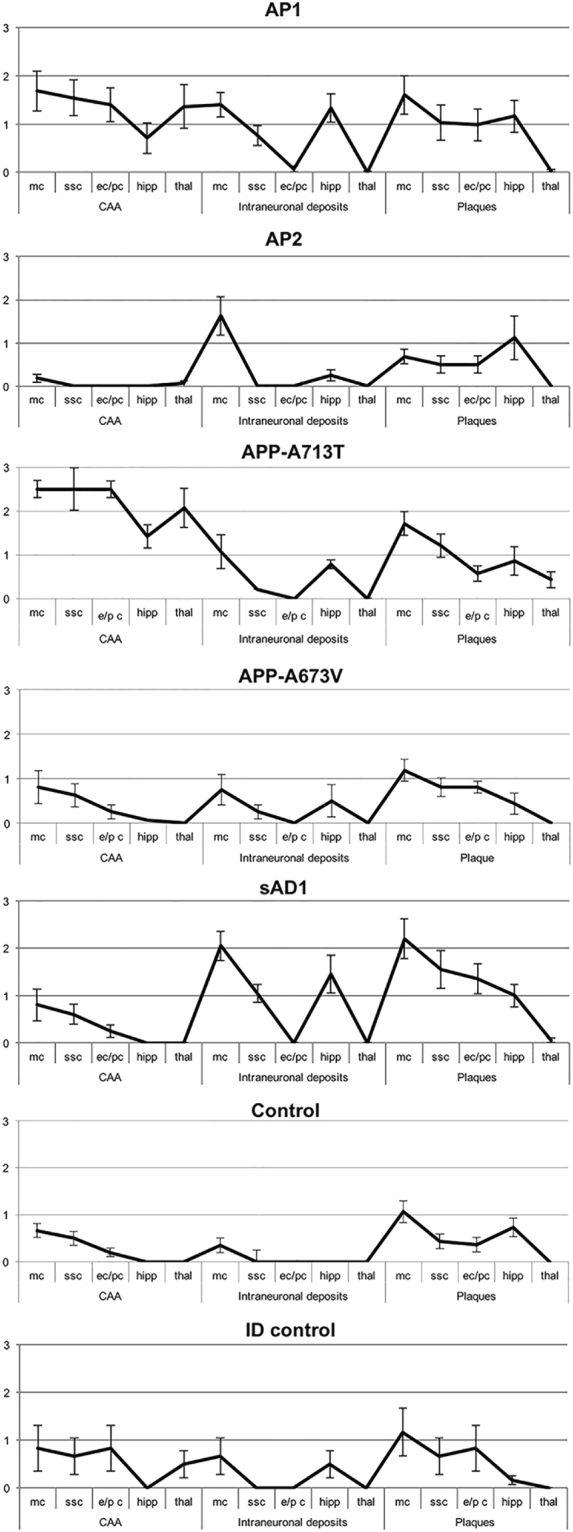
Lesion profiles in mice inoculated with human brain extracts from AD patients. CAA, intraneuronal Aβ immunostaining and amyloid plaques were used to build up lesion profiles of the disease in mice. Control = untreated age-matched mice. mc = motor cortex; ssc = somato-sensory cortex; ec/pc = enthorinal cortex/piriform cortex; hipp = hippocampus; thal = thalamus. Immunohistochemical study was performed with 4G8 antibody. Quantification of 4G8 immunostaining was calculated by ‘plaque count’ method and expressed in each profile as a mean ± SEM of the values obtained in animal groups (n = 8) injected with human brain extracts of each molecular profile: AP1-AP2 subgroups and APP_A673V_ (fAD1), APP_A713T_ (fAD2) and sAD1 case. Quantification by ‘plaque count’ was carried out using a scale ranging from 0 to 5 by light microscopy. The study was performed using “NIS-elements” software”.

**Figure 7:**
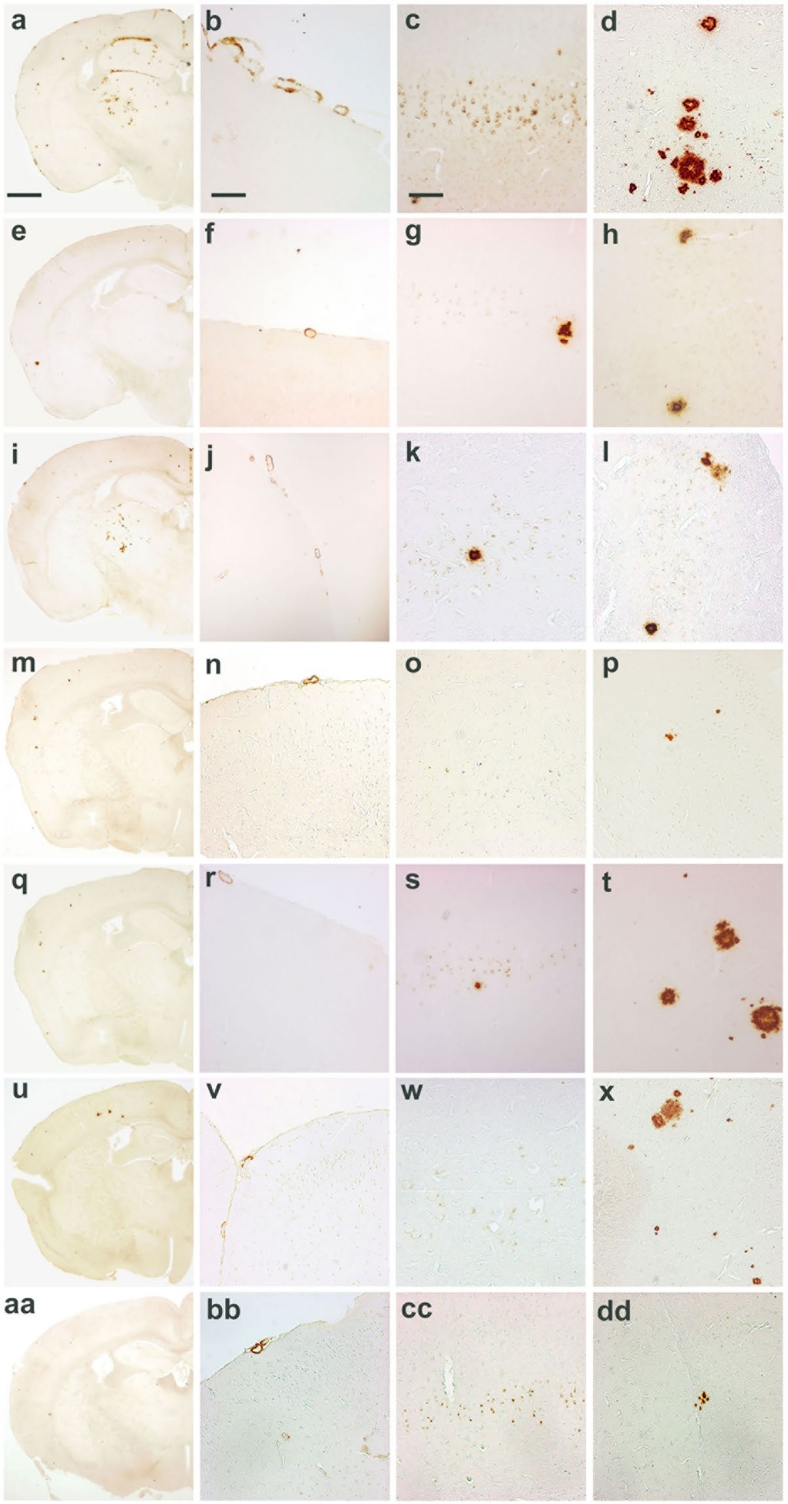
Amyloid burden in mice injected with human brain extracts from distinct molecular profiles of Alzheimer’s disease. Mice inoculated with an Alzheimer brain homogenate from AP1 subgroup (**a**–**d**), AP2 subgroup (**e**–**h**), APP_A713T_ (fAD2) (**i**–**l**), APP_A673V_ (fAD1) (**m**–**p**), sAD1 case (**q–t**) and control groups, i.e. age-matched noninjected mice (**u**–**x**) and mice injected with Aβ-immunodepleted brain extracts (**aa–dd**). (**a,e,i,m,q,u,aa**) Amyloid deposits, scale bar 0,5 mm. (**b,f,j,n,r,v,bb**) Congophilic amyloid angiopathy, 300 µm. (**c,g,k,o,s,w,cc**) Intraneuronal Aβ immunoreactivity, scale bar 120 µm. (**d,h,l,p,t,x,dd**) Amyloid plaques, scale bar 120 um. Immunostaining with 4G8 antibody.

Finally we assessed whether different molecular subgroups of AD have different abilities to propagate the pathological process *in vivo*, when injected in animal models^30^. We found that moApp^0/0^/APP23^+/−^, intra-cerebrally inoculated at six months of age with brain homogenates selected among the molecular subgroups previously identified, developed brain amyloidoses with distinctive disease profiles as to morphology, regional distribution of amyloid deposits, and preferential parenchymal or vascular Aβ deposition (Figs 6 and 7), indicating that the phenotypic diversity of human pathology can be maintained upon transmission to mice, even if without a close replication of the features characterizing human donor brains.

Interestingly, the severity of neuropathological changes induced in mice by the brain extracts belonging to different AD subgroups was different suggesting that Aβ seeds contained in human brain homogenates have physicochemical properties affecting their ability in propagating amyloidosis in animal models (Figs 6 and 7). Indeed, AP1 subgroup was more effective than AP2 subgroup in inducing and propagating the disease (Fig. 6). The brain extracts from the two APP-mutated patients (AP3 subgroup) showed relevant dissimilarities and were separately illustrated in Figs 6 and 7. Seeds from the sAD1 case were also particularly aggressive (Fig. 6).

Human brain extracts from AP1 induced an amyloidosis characterized by intense amyloid burden, with amyloid aggregates consisting of small sized and diffuse plaques, strongly associated with diffuse CAA and intraneuronal Aβ immunoreactivity localized in hippocampus and neocortex (a-d panel in Fig. 7). In this group we observed abundant Aβ-positive CAA with plaques within the thalamus, a peculiarity reported by Watts *et al*.^31^ in APP23 mice inoculated with brain extracts derived from patients carrying the *APP* ‘Artic’ mutation.

Inoculation of AP2 samples resulted in a weaker and diffuse amyloid deposition, with faint CAA and intracellular immunostaining for Aβ. The thalamus was not involved (e–h panels in Fig. 7).

Amyloidosis induced by APP_A713T_ (fAD2) brain samples was characterized by low amyloid burden, high content of CAA, low amount of intraneuronal Aβ immunostaining. Also in this group the lesion profile showed a thalamic deposition of Aβ in vessel walls and, to lesser extent, in parenchymal deposits (i–l panels in Fig. 7). However, the pathologic changes in thalamus were less intense than in mice injected with AP1 brain extracts. APP_A673V_ (fAD1) caused only a faint amyloidosis (m–p panels in Fig. 7).

Injection of brain extract from the sAD1 patient was associated with the highest amount of intraneuronal immunostaining for Aβ especially present in hippocampus and motor cortex, diffuse parenchymal amyloid deposits showing large size, and less consistent CAA sparing thalamus (q-t panels in Fig. 7).

Intracerebral injection of human brain samples depleted of Aβ seeds by immunodepletion did not modify the spontaneous amyloidogenesis of moApp^0/0^/APP23^+/−^ mice (bottom panel in Fig. 6 and aa-dd panels in Fig. 7), confirming the view supported by previous reports^26,32,33^ that Aβ seeds are necessary to accelerate amyloidosis in the host. Inoculation of human extracts into nontransgenic littermate control mice was not associated with the development of amyloidosis. Injection of brain homogenate from a nondemented control did not modify the spontaneous amyloidogenesis of transgenic mice (data not shown). Finally, second passage inoculations of moApp^0/0^/APP23^+/−^ mice were carried out. The resultant amyloidosis generated in animals retained the pathologic features induced by first passage injections in each experimental group, but showed a global attenuation of the severity of neuropathologic changes (data not shown), in line with previous studies demonstrating the resilience of Aβ seeds^34^.

We also measured the levels of Aβ40 and Aβ42 in the insoluble fraction of brain homogenates from mice injected with human brain extracts. The results substantially confirmed the differences revealed by the neuropathological studies showing higher Aβ levels in mice injected with AP1 subgroup, APP_A713T_ and sAD1 patients. The AP2 subgroup and the APP_A673V_ case showed lower amount of Aβ. The immunodepleted controls had Aβ levels similar to non-injected mice (Fig. S4).

## Discussion

Phenotypic heterogeneity of AD is a very complex phenomenon whose molecular basis is still largely unexplored. Recently, a correlation between Aβ fibril structure and variations in AD phenotype has been demonstrated by solid-state nuclear magnetic resonance measurements on Aβ40 and Aβ42 fibrils prepared by seeded growth from AD brain extracts. These studies indicated that there is a qualitative difference between Aβ aggregates in the brain tissue of patients with AD and that this difference may result in the generation of distinct disease phenotypes, in analogy to distinct prion strains that are associated with different phenotypes of prion diseases^35^.

Our study focused on one of the molecular aspects which might lead to the generation of differences in the structural properties of Aβ assemblies that in turn may be involved in generation of distinct disease phenotypes. We found that: (i) amyloid-β is generated starting from different mixtures of Aβ peptides that participate in the amyloid biochemical composition; (ii) different subtypes of amyloid-β might undergo distinct aggregation pathways, generating Aβ assemblies that display different toxic properties, depending on the time-course of the lag and growth phases in their aggregation kinetics; (iii) amyloid subtypes also show variability in their seeding activity on monomeric Aβ1-42, which depends on the seed-substrate affinity during the polymerization process; (iv) aggregates from different amyloid-β subtypes have diverging resistance to degradation by proteases, giving rise to assemblies that can be variably stable and toxic *in vivo*; (v) amyloid subtypes have differential ability toward anticipating or accelerating amyloidogenesis in animal models of AD and generate amyloidosis showing distinct features in mice injected with human brain extracts.

Moreover, it’s conceivable that unknown environmental elements in the host brain tissue may be involved in modeling the pathologic changes of human brain-induced amyloidosis in mice, so explaining why the mouse pathology is not the simple replication of the human pathology.

The finding that genetic forms of the disease (i.e., those associated with *APP* mutations) have a molecular profile distinct from sporadic cases is not surprising, but our data support the view that molecular heterogeneity is a feature of sporadic forms too^14,36–38^.

Interestingly, amyloid profile enriched in Aβx-42 peptides (i.e., AP1) showed fast aggregation kinetics, strong seeding abilities, high resistance to proteolysis and aggressiveness in animal models. However, the high amount of the longest Aβ isoform (i.e., Aβ1-42) in this subgroup cannot fully explain the aggressiveness of AP1 Aβ seeds. Indeed, sAD1 case, which is characterized by a prevalence of Aβx-40 peptides, actually showed the most aggressive molecular phenotype. On the other hand, Aβ seeds from patients carrying *APP* mutations (AP3 in our molecular grouping) displayed a heterogeneous behavior, one (APP_A713T_) being more aggressive, the other (APP_A673V_) showing only faint aggregation and seeding abilities, and inducing weak amyloid deposition in mice injected with its brain extracts. These last findings are in line with the results of several studies indicating that the heterologous interaction between wt and A673V-mutated Aβ peptides results in inhibition of Aβ polymerization^39,40^.

These results suggest that seeding and aggregation properties, resistance to proteolysis and aggressiveness of the neuropathological phenotype induced in animal models are caused by the differences in the Aβ content that typify each AD subgroup.

Overall these data led to the recognition of 2 main distinct molecular profiles of the disease in the series of sporadic AD cases included in our study and supported a molecular clustering of AD based on the structural and functional properties of the ‘amyloids’ identified in each subgroup.

The overall data obtained by RT-QuIC assays supported the view that the differences in the aggregation profiles are not simply due to the quantities of Aβ40 or Aβ42 in the substrate source of brain tissue (i.e., soluble fraction of brain homogenates), but are reasonably influenced by the nature (i.e., biochemical content) of the initial seeds which trigger Aβ polymerization as well as by the affinity between seed and substrate. Indeed, sAD1, which contains much more Aβ40 than Aβ42 species, greatly promotes Aβ1-42 polymerization even more than the AP1 subgroup that is much more enriched in Aβ42 isoforms.

The data on sAD1 case further support our hypothesis that some differences in the *sporadic* AD subgroups may be related to intrinsic properties of the peptides involved into the aggregation kinetics. Based on this hypothesis, a certain mixture of Aβ peptides may follow an aggregation pathway distinct from another mixture, leading to cerebral amyloidosis with distinctive characteristics. The peculiar features displayed by the sAD1 case also suggest that other variant AD subgroups may exist.

Intriguingly, two very recent papers suggested a structural variability of Aβ aggregates in both sporadic and familial AD patients, supporting our hypothesis on the existence of distinct AD molecular subtypes^41,42^.

Phenotypic differences in AD stem from the interaction of a series of elements including genes that modulate the risk of developing the disease (such as *ApoE*) and less known environmental factors^43,44^ which, together with predisposing components, may write the history of the disease as different clinical and pathological traits. In our study, we did not find a correlation between *ApoE* genotype and amyloid profile. Further studies on larger cohorts of patients and controls are needed to address this point.

Use of transmission studies for the identification and characterization of different forms of the disease is a yet poorly explored approach in the field of AD^31^. It has been successfully used for prion diseases to unveil the existence of different prion strains responsible for different prion-related pathologies^45^. Our study stems from previous evidence that amyloid deposition can be induced by injection of human brain extracts into animals which develop a cerebral amyloidosis involving also brain areas far from the injection site^26,33,46^. These data suggest that the typical brain abnormalities associated with AD can be induced by a prion-like mechanism based on the propagation of protein misfolding across brain tissue. Moreover, phenotypic variability is a salient feature of prion diseases where it is clearly sustained by the existence of distinct subtypes of prions^47,48^. These observations remind to the general concept of prion-like induction and spreading of pathogenic proteins that has been recently expanded to include aggregates of tau, α-synuclein, huntingtin, superoxide dismutase-1, and TDP-43, which characterize several human neurodegenerative disorders such as frontotemporal lobar degeneration, Parkinson’s/Lewy body disease, Huntington’s disease and amyotrophic lateral sclerosis^46,49,50^. Noteworthy, all these diseases can present under different phenotypes, but the role of the disease-related misfolding proteins in the generation of their phenotypic variability has not yet been explained^51,52^.

Misfolding of PrP is the result of the intrinsic property of the prion protein to adopt different conformations. and generate different conformers of the protein, which can give raise to different subtypes of prionopathies^53^. Our data suggest the view that AD exists as distinct molecular phenotypes as a result of differences in the mixture of peptides participating in Aβ assemblies.

Noteworthy, the variances in the amyloid composition offer a sort of bar code to identify different molecular AD subgroups.

We recently demonstrated that the preferential accumulation of some Aβ fragments in amyloid was paralleled by a reduction of the very same fragments in the CSF of AD cases. This offers grounds to the detection of distinct AD subgroups by the analysis of Aβ profile in CSF of AD patients^54^.

The comprehension of the molecular machinery responsible for the phenotypic diversity in AD is still at the beginning of its history, but it is becoming an urgent need, considering the emerging evidence that the different responsiveness to pharmacological treatments among AD cases could be due at least in part to the existence of distinct subgroups of the disease^55,56^ diverging for their clinico-pathological, biochemical profiles, and pathogenic pathways too. The relevance of molecular heterogeneity of Aβ assemblies in the generation of specific clinico-pathological AD phenotypes needs further studies in larger cohorts of patients. However, our study may help to understand the molecular bases of disease heterogeneity and design more appropriate therapies based on recognition of different target phenotypes.

## Materials and Methods

### Selection and neuropathological characterization of AD cases

Informed consent was obtained from all individual participants included in the study. All procedures were in accordance with the 1964 Declaration of Helsinki and its later amendments and were approved by Ethical Committee of Carlo Besta Neurological Institute. Characterization of AD cases (n = 24) was primarily based on their neuropathological profiles and in consideration of burden, morphology and distribution of amyloid plaques, relative percentage of parenchymal and vascular deposits, immunoreactivity for a panel of antibodies to epitopes of different Aβ domains^7^ (see ‘Materials and Methods’ section in *Supplementary Information* for details). The *ApoE* genotype was determined in all cases. Genetically inherited AD cases were identified as fAD1 to fAD4. Sporadic cases were identified as sAD1 through sAD20. See Table S1 in *Supplementary Information* for details.

### Amyloid extraction

#### Complete protocol

Amyloid was purified from frozen brain tissue of five AD cases following a method previously described for PrP amyloid^57^ and applied to β-amyloid. In particular, 8 g of frontal cortex were serially homogenized in 9 volumes of buffer A (10 mM Tris-HCl, Sigma-Aldrich, St. Louis, MO pH 7.5, 150 mM NaCl, Sigma-Aldrich, St. Louis, MO, 1% Triton X-100, Amresco, Solon, OH, aded with Complete Protease Inhibitors cocktail, Roche, Mannheim, Germany), buffer B (10 mM Tris-HCl pH 7.5, 0.6 M KI, Sigma-Aldrich, St. Louis, MO, 0.5% Triton X-100, Complete Protease Inhibitors cocktail) and buffer C (10 mM Tris-HCl pH 7.5, 1.5 M KCl, Sigma-Aldrich, St. Louis, MO, 0.5% Triton X-100, Complete Protease Inhibitors cocktail). After each step, the homogenate was centrifuged at 10,000 xg for 40 minutes at 4 °C. The pellet was washed four times in buffer D (50 mM Tris-HCl, 150 mM NaCl, pH 7.5), centrifuged at 55,000 xg for 40 minutes at 4 °C, and digested with Collagenase Type1 at 37 °C for 18 hours. After centrifugation at 70,000 xg for 1 hour at 4 °C, the pellet was washed three times in 50 mM Tris-HCl pH 7.5, loaded on a discontinuous sucrose gradient (1.0, 1.2, 1.4, 1.7, 2.0 M sucrose, Sigma-Aldrich, St. Louis, MO, in 10 mM Tris-HCl pH 7.5) and centrifuged at 130,000 xg for 2 hours at 20 °C. Each interface was collected, washed three times in buffer D and centrifuged at 55,000 xg for 30 minutes at 4 °C. Amyloid was extracted with 80% formic acid, Sigma-Aldrich, St. Louis, MO, dried and re-suspended in H_2_O for further analysis.

#### Simplified protocol

A simplified amyloid extraction protocol was applied to the same five AD cases. Briefly, three hundred mg of frontal cortex were homogenized in 9 volumes of 10 mM Tris-HCl, pH 7.5, 0.5% Triton X-100 added with Complete Protease Inhibitors cocktail using a manual Dounce homogenizer, sonicated for 2 minutes using a Ultrasonic homogenizer Sonopuls-series HD2070 and centrifuged at 3,000 xg for 5 minutes at 4 °C. The supernatant was loaded on a discontinuous sucrose gradient (1.0, 1.4, 1.8 M sucrose in 10 mM Tris-HCl pH 7.5) and centrifuged at 130,000 xg for 2 hours at 20 °C. Each interface was collected, washed three times in 50 mM Tris-HCl, 150 mM NaCl, pH 7.5 and centrifuged at 55,000 xg for 30 minutes at 4 °C. The pellet was treated with 80% formic acid, dried and re-suspended in H_2_O for further analysis.

No substantial differences were observed between amyloid samples obtained by full and simplified sucrose-gradient fractionation protocols (data not shown). So, we used the simplified procedure to extract amyloid from all other AD cases and controls.

### Immunoproteomic analyses

The immunoproteomic assay for Aβ isoforms detection was performed as previously reported^58^, with minor modifications. Briefly, 3 µl of a 0.125 mg/ml monoclonal antibody solution (6E10 and 4G8, Covance, Dedham, MA) was incubated for 3 h at room temperature in a humidity chamber to allow covalent binding to the PS20 ProteinChip Array (Bio-RAD Laboratories Inc., Hercules, CA). Unreacted sites were blocked for 1 h at room temperature with 0.4 M Tris-HCl, pH 8.0, in a humidity chamber. Each spot was washed three times with PBS containing 0.5% (v/v) Triton X-100 then twice with PBS alone. Spots were coated with 5 µl of sample and incubated at 4 °C overnight in a humidity chamber before being washed three times with PBS containing 0.1% (v/v) Triton X-100, twice with PBS alone and finally with deionized water. 1.2 µl of α-cyano-4-hydroxy cinnamic acid (Bio-RAD Laboratories, Inc., Hercules, CA) was added to each spot and mass identification was performed using the ProteinChip SELDI System, Enterprise Edition (Bio-RAD Laboratories, Inc., Hercules, CA). The different amyloid profiles characterizing each AD subgroup were identified taking into account the relative percentage of the different Aβ isoforms detected by SELDI-TOF MS.

### Brain homogenates

Brain homogenates were prepared as previously described^59^. Briefly, 200 mg of frontal cortex were homogenized in 5 volumes of 20 mM Tris-HCl, pH 7.5, 140 mM NaCl, added with Complete Protease Inhibitors cocktail and Phosphatase Inhibitors Cocktail 2 (Sigma) using a manual Dounce homogenizer and ultracentrifuged at 100,000 xg for 1 hour at 4 °C. The supernatant was collected, aliquoted and stored at −80 °C as the S1fraction. The pellet was re-homogenized in 1% Chaps, 1% Deoxycholate, 0.2% SDS, 140 mM NaCl, 10 mM Tris-HCl, pH 7.5, added with Protease and Phosphatase Inhibitors and ultracentrifuged at 30,000 xg for 30 minutes at 4 °C. The supernatant was aliquoted and stored at −80 °C as the S2 fraction; the pellet was homogenized in 2% SDS, 20 mM Tris-HCl, pH 7.5, 140 mM NaCl and ultracentrifuged at 30,000 xg for 30 minutes at 4 °C. The supernatant was saved as the S3 fraction and stored at −80 °C; the pellet was extracted in 4% SDS, 8 M Urea (P3 fraction). The total proteins amount was measured in each fraction by BCA Protein Assay kit (Pierce). Immunodepletion was carried out by using Protein G Mag Sepharose beeds (GE Healthcare) and a mixture of 4G8 and 6E10 antibodies.

### Thioflavin T aggregation assay

5 microliters of brain homogenates’ soluble fractions (S1) were diluted in 100 mM Tris-HCl pH 7.5, 5 μM ThT, and transferred in triplicate into wells of a black, clear bottom, 96-well microplate (Nunc). The plate was incubated at 25 °C into a BMG Fluostar Optima Microplate Reader (BMG Labtech). Every 59 minutes the plate was shaked for 1 minute and the fluorescence was measured. For the comparison of aggregation kinetics among Aβ extracted from different AD brains, we considered the slope of the curve described by fluorescence values at different time points.

A preliminary step in the ThT assays was used to test the effects of the initial quantity of soluble Aβ in AD brain extracts on the shape of aggregation curves. To this end, 5 or 10 microliters of brain homogenates’ soluble fractions (S1) of some human brain samples were analyzed by ThT assay and showed overlapping profiles.

### Real-Time Quaking-Induced Conversion (RT-QuIC) assay

For these assays, a previously described protocol^60^ - not yet fully validated – was used. It was slightly modified in order to comply with the analysis of brain samples.

5 microliters of brain homogenates’ S1 fractions were used as *seeds* and diluted in 100 mM Tris-HCl pH 7.5, 5 μM ThT, 4 μM synthetic Aβ1-42_WT_ or Aβ1-42_A2V_. The reactions were transferred in quadruplicate into wells of a black, clear bottom, 96-well microplate (Nunc). The plate was incubated at 37 °C into a BMG Fluostar Optima Microplate Reader (BMG Labtech), and shaken every other minute. Fluorescence was measured every 15 minutes. The same protocol was used to test the seeding ability of brain extracts on Aβ1-40_WT_, except for Aβ1-40 concentration (10 μM) and incubation temperature (30°).

### Sensitivity to PK digestion

For proteinase K (PK) digestion, 5 micrograms (or 1 microgram for patients carrying A673V and A713T APP mutations) of total proteins from P3 fractions were digested with increasing levels (between 0 and 100 µg/ml) of PK for 1 hour at 37 °C. PK digestion was blocked by adding Bolt LDS Sample Buffer (Invitrogen) and Bolt Sample Reducing Agent (Invitrogen) and incubating 10 minutes at 70 °C; the samples were analyzed by Western blot.

#### Western blot

Samples were loaded on Bolt 4–12% Bis-Tris poliacrylamide gels (Invitrogen), transferred to PVDF and immunoblotted with 4G8 (Signet) diluted 1:1000. The membranes were then sequentially incubated with biotin-goat anti-mouse (Invitrogen) and Streptavidin-horseradish peroxidase conjugate (GE Healthcare) and revealed using ECL Prime (GE Healthcare). The signal intensity was quantified by densitometry using the software Quantity One (BioRad).

### Transmission studies

The experimental procedures using mice were carried out in accordance with the veterinary office regulations established by both the Council of Europe Convention ETS123 (European Convention for the Protection of Vertebrate Animals used for Experimental and Other Scientific Purposes; Strasbourg, 18.03.1986) and the European directive 2010/63 transposed in Italian D.L.vo 26/14. The study was approved by Italian Ministry of Health (approval number 1219/2015-PR).

Whole brain homogenates from AD cases belonging to the different molecular subtypes recognized by biochemical studies (AP1-AP3 and the sAD1 case) and two controls (one age-matched nondemented subject and one AD brain homogenate deprived from Aβ by immunodepletion with anti-Aβ antibodies) were prepared as follows. Samples of frontal cortex were homogenized in 9 volumes of sterile 1× PBS using a manual Dounce homogenizer, sonicated for 15 seconds using a Ultrasonic homogenizer Sonopuls-series HD2070 and centrifuged at 3,000 xg for 5 minutes at 4 °C. The supernatants were collected, aliquoted and stored at −80 °C until inoculation in APP23 mice (carrying the double Swedish human APP mutation), knock-out for endogenous App (moApp^0/0^/APP23^+/−^), chosen to avoid the interference of murine App in the propagation of the disease. See *Supplementary Information* for details.

### Statistical analysis

Student t-test was used to compare amyloid burden in immunohistochemical studies on human brains and on brains from mice inoculated with human cerebral homogenates. The densitometric data obtained from the quantification of Western Blot for the study of PK resistance were compared by two-ways ANOVA. Kruskal-Wallis followed by Dunn’s multiple comparison test was used to compare Aβ40 and Aβ42 levels measured by ELISA in the insoluble fractions of injected mice. Two tailed P value less than 0.05 was considered statistically significant. All calculations were performed using GraphPad Prism 5.

### Data availability statement

The datasets generated during and/or analyzed during the current study are available from the corresponding author on reasonable request.

### Ethical approval

All procedures performed in studies involving human participants were in accordance with the ethical standards of the institutional and/or national research committee and with the 1964 Helsinki declaration and its later amendments. All applicable international, national, and/or institutional guidelines for the care and use of animals were followed.

## Electronic supplementary material

Supplementary Information
