# Supplementary material for: Molecular subtypes of Alzheimer’s disease
Source: Sci Rep. 2018 Feb 19;8:3269. doi: 10.1038/s41598-018-21641-1 (PMC5818536; doi:10.1038/s41598-018-21641-1)
Supplement: Supplementary file 1 — Supplementary Information [file 41598_2018_21641_MOESM1_ESM.docx]

**Supplementary Information**

**Molecular subtypes of Alzheimer’s disease**

Giuseppe Di Fede^1^, Marcella Catania^1^, Emanuela Maderna^1^, Roberta Ghidoni^2^, Luisa Benussi^2^, Elisa Tonoli^2^, Giorgio Giaccone^1^, Fabio Moda^1^, Anna Paterlini^2^, Ilaria Campagnani^1^, Stefano Sorrentino^1^, Laura Colombo^3^, Adriana Kubis^1,4^, Edoardo Bistaffa^1^, Bernardino Ghetti^5^, Fabrizio Tagliavini^1*^.

^1^IRCCS Foundation ‘‘Carlo Besta’’ Neurological Institute, Milan, Italy

^2^Molecular Markers Laboratory, IRCCS Istituto Centro San Giovanni di Dio - Fatebenefratelli, Brescia, Italy

^3^Department of Molecular Biochemistry and Pharmacology, IRCCS-Istituto di Ricerche Farmacologiche “Mario Negri”, Milan, Italy

^4^Department of Toxicology, Wroclaw Medical University, Wrocław, Poland

^5^Department of Pathology and Laboratory Medicine, Indiana University, Indianapolis, Indiana, USA

*Corresponding author:* Fabrizio Tagliavini, IRCCS Foundation “Carlo Besta” Neurological Institute, Via Celoria 11, 20133 Milan, Italy; phone: +39 02 2394 2335; email: [fabrizio.tagliavini@istituto-besta.it](mailto:fabrizio.tagliavini@istituto-besta.it)

**MATERIAL AND METHODS**

**Selection and neuropathological characterization of Alzheimer’s disease cases**

***Case series***

The study was performed examining the brains of twenty four patients with sporadic (sAD, n=20) and familial (fAD, n=4) Alzheimer’s disease (AD) associated with the following mutations: APP_A673V_^1-3^, APP_A713T_^4^, PS1_P117A_^5^ and PS2_A85V_^6^. Nine non-demented age-matched controls were included in the study. Neuropathological assessment was carried out according to the international guidelines for the neuropathology diagnosis of AD^7,8^.

***Histology and Immunohistochemistry***

For each case, the left cerebral hemisphere, the cerebellum and the brainstem were fixed in 10% formalin. Coronal slices of the left cerebral hemisphere were dehydrated in graded ethanol, cleared in xylene, embedded in paraffin, cut into 12-μm-thick sections using a Reichert-Jung polycut E microtome (Leica Microsystem). Routine examination was carried out on sections stained with hematoxylin–eosin, cresyl violet for Nissl substance, Heidenhein–Woelcke for myelin, thioflavine S for amyloid and Bodian silver impregnation.

Immunohistochemistry was performed using antibodies recognizing Aβ and tau proteins as well as markers of secondary lesions usually observed in AD (including astrocytosis and microglial activation). Before Aβ immunostaining, the sections were pre-treated with formic acid (80 %, 60 min). The anti-Aβ antibodies used for the study were: a monoclonal antibody reactive to amino acid residues 17–24 of Aβ (clone 4G8, 1:2000, Covance) that is widely used in routine diagnostic procedures ^9^; monoclonal antibodies reactive to the C-terminus of Aβ, specifically recognizing Aβ40 (clone 11A50-B10, 1:1000, Covance), Aβ42 (clone 12F4, 1:500, Covance) or Aβ38 peptides (clone BA1-13 and 7-14-4, 1:200, Covance). An antibody against phosphorylated tau (clone AT8, 1:300, Innogenetics) was used to detect neurofibrillary changes. Immunohistochemistry for glial fibrillary acidic protein and CR3–43 as marker of activated microglia was carried out using two specific antibodies (polyclonal anti-GFAP antibody, 1:800, and monoclonal anti-human HLA-DP, DQ, DR Antigen, 1:200, DakoCytomation, respectively). The immunoreactions were visualized by the EnVision Plus/horseradish peroxidase system for rabbit or mouse immunoglobulins, using 3-30-diaminobenzidine as chromogen (DakoCytomation).

***Staging protocols***

Each brain region was evaluated according to the guidelines of ‘Consortium to Establish a Registry for Alzheimer’s Disease (CERAD) Cases’ ^7,10,11^. To analyze amyloid-related pathology, we assigned a plaque density score (ranging from 0 to 3) determined by estimating the mean amyloid burden in different brain areas ^7^. Amyloid vascular deposits were estimated according to Vonsattel staging system for CAA, using a scale ranging from 1 to 4 arbitrary units ^12^. Neurofibrillary pathology was assessed according to Braak staging system (I to VI) ^13,14^.

***Amyloid burden quantification***

Sections immunostained with 4G8 were processed on a computer-assisted image analysis system, consisting of a light microscope (Nikon Eclipse E800, Nikon) equipped with a digital camera (Nikon DXM 1200) connected to a personal computer running NIS Elements software (Nikon). The following brain regions were examined: frontal cortex (Supplementary Table 1), anterior cingulate cortex, temporal cortex, insular cortex, parietal cortex, hippocampus, striatum, occipital cortex. The areas were studied as part of the entire coronal section. Images of each region of interest in thick sections were captured (4x), and a threshold of optical density that discriminated staining from background was obtained. Manual editing eliminated artifacts. The total ‘‘amyloid burden’’ was defined as the mean of objects count in standard ROI (dimensions 68.5 x 54.9 μm), immunostained for 4G8, over 3 measurements (object count) selected according to a random sampling model in each cortical areas^15^. Image analysis software estimated for each microscopic field the total Aβ load as the mean value of object count^15^ and area fraction^16^ for each cortical area.

Semiquantitative analysis of the immunostaining with antibodies against 4G8, AT8, Aβ38, Aβ40 and Aβ42 was carried out in depth in frontal cortex (i.e., the same brain area used for biochemical studies), temporal and parietal cortex using the following evaluation scale: -, -/+, +, ++. We identify three types of plaques related to their morphology: diffuse, neuritic and cotton wool plaques. Finally, we calculated the dispersion index (DI) as the ratio between the number of Aβ positive structures and the area fraction for each cortical field. Following this method, microscopic fields characterized by a low number of large-size Aβ deposits display low DI, while areas showing myriads of tiny Aβ deposits have high DI values^17^ (Supplementary Table 1).

**Neuropathological assessment of cerebral amyloidosis in mice**

For transmission studies, we used APP23 mice (carrying the double Swedish human APP mutation), knock-out for endogenous App (moApp^0/0^/APP23^+/-^), chosen to avoid the interference of murine App in the propagation of the disease. A longitudinal neuropathological study on these animals revealed that this transgenic (tg) line develops amyloid deposits at the age of 9 months. Presence of amyloid deposits throughout the brain is observed at the age of 13-15 months. Eight experimental groups (n=9) have been inoculated at 6 months of age, in a pre-amyloid phase, with brain homogenates from the eight AD patients above described. Tg mice challenged with mock inocula, i.e. non-AD brain extracts (five animals) or AD brain homogenates deprived of Aβ seeds by immunoprecipitation with 4G8 antibody against amyloid protein (seven animals) have been used as control groups. Moreover, the same AD brain extracts were used to inoculated non-transgenic mice. Mice were inoculated by stereotaxic surgery in the hippocampus of the right hemisphere with 2.5 μl of human brain homogenate following these coordinates: 2.5 mm caudal, 2.0 mm lateral, 1.8 mm depth. Three or five months after inoculation, mice underwent neuropathological and biochemical assessment. We also carried out second passage inoculations by injecting whole brain homogenates from previously human brain-injected mice into 5 month-aged moApp^0/0^/APP23^+/-^ animals following the same protocol used for first passage.

The assessment of β-amyloid burden in tg mice inoculated with human brain homogenates as well as in mice subjected to second passage inoculations was obtained by the analysis of (i) area fraction (% of plaques detected by immunohistochemistry with 4G8 antibody) and (ii) object count (number of amyloid plaques in selected ROI of standard dimensions, 1870 x 835 μm) in six different brain areas: motor cortex (*mc*), somato-sensory cortex (*ssc*), enthorinal-piriform cortex (*ec/pc*), hippocampus (*hipp*) and thalamus (*thal*). The study was performed using “NIS-elements” software.

For histopathological analysis of moApp^0/0^/APP23^+/-^ injected with human brain homogenates, coronal slices of the hemibrains were embedded in paraffin and cut (7 μm); sections were de-waxed in xylene (5 min, two times) and hydrated through serial alcohols (100%, 2 min., two times; 95%, 2 min.; 90%, 80% 70%, 2 min) to water. Brain tissues were stained by hematoxylin and eosin and cresyl violet for Nissl substance.

After formic acid (80%) pre-treatment to abolish intraneuronal immunoreactivity, sections were incubated over-night with anti-Aβ antibody (4G8, 1:4000, Covance). The immunolabeling with the primary antibody was detected using a biotinylated secondary antibody followed by horseradish streptavidin peroxidase. The complexes were visualized with DAB and the sections were dehydrated through serial alcohols and xylene. Immunostaining with antibodies against glial fibrillary acidic protein (1:600; DakoCytomation) and IBA1 (1:500; Abcam) was also performed.

Amyloid deposition was assessed in mouse brain using Aβ immunostaining (4G8 antibody) in different cortical areas: motor cortex (*mc*), somato-sensory cortex (*ssc*), enthorinal-piriform cortex (*ec/pc*), hippocampus (*hipp*) and thalamus (*thal*). The assessment was made in two adjacent sections of the same brain area ^15^. Quantification of Aβ load was calculated following two parallel approaches: the ‘plaque count’ ^15^ and the ‘area fraction’ methods ^16,18,19^.

Quantification by ‘plaque count’ was carried out using a scale ranging from 0 to 5 by light microscopy. The total amyloid burden was defined as the mean of objects count in standard ROI (dimensions: 1870 x 835 μm), immunostained for 4G8, over 3 measurements in each cortical area.

Area fraction in the same areas was calculated using an image analysis software (NIS-elements-Nikon) and expressed as the percentage of space occupied by plaques identified by 4G8 antibody in each selected ROI. Images (4x) of each region of interest in thick sections were captured, and a threshold of optical density that discriminated staining signal from background was obtained. Manual editing eliminated artifacts.

**ELISAs**

The left hemibrains of injected mice were homogenized in 7 volumes of 20 mM Tris-HCl pH = 7.5 added with Complete Protease Inhibitors cocktail (Roche) using a manual Dounce homogenizer and ultracentrifuged at 100,000 xg for 1 hour at 4°C. The supernatants were collected as the soluble fractions; the pellets were extracted in 70% Formic Acid, neutralized with 20 volumes of 1M Tris and stored as the insoluble fractions. Aβ40 and Aβ42 were measured in duplicate in the insoluble fractions using commercially available ELISA kits (Human Amyloid β40 and β42 ELISA, Millipore). The total proteins amount was measured by BCA Protein Assay kit (Pierce); Aβ40 and Aβ42 levels were normalized to total proteins.

**RESULTS**

***Assessment of Amyloid-related pathology in the study population***

Demographic and neuropathological features of the cohort are summarized in Table S1. We used immunohistochemistry with a panel of antibodies against Aβ and semiquantitative evaluations of the amyloid burden to detect differences in the presentation of amyloid-related pathology in a series of sporadic and familiar AD patients. Amyloid-β density, size and shape of amyloid deposits, brain regional distribution and relative amount of vascular, parenchymal and intracellular Aβ deposition were analyzed.

The study revealed that amyloid deposition can consistently differ among AD patients. Differences include both inter-individual variability (Table S1) and brain regional variability (data not shown). However, if we consider the same cortical area (i.e., frontal cortex), immunoreactivity for 4G8 antibody spanned from ‘weak’ (AD5 in table S1) to ‘diffuse and strong’ (fAD3); Aβ42 immunostaining was remarkable in the PS1-P117A patient (fAD3), where it was associated to a ‘weak’ immunoreactivity for Aβ40 and Aβ38; immunostaining for Aβ38 was especially evident in the APP-mutated group (fAD1 and fAD2); the sAD1 case was characterized by a prevalence of Aβ40 over Aβ42 immunostaining. So, the different immunoreactivity of amyloid deposits to antibodies whose epitopes span over distinct domains of Aβ suggest that the amyloid aggregates accumulating in the brain of AD cases may have a different content of Aβ peptides. Moreover, CAA was more represented in APP-mutated cases and in the sAD1 patient.

The variability of amyloid burden and the differences in size and shape of amyloid deposits that we found in the frontal cortex from distinct patients are findings consistent with the view that a neuropathological heterogeneity occurs not only among genetically inherited forms of AD but also among sporadic cases.

Finally, no statistically significant correlation was found between disease duration or age at onset and molecular patterns emerged in the cohort of patients analyzed in this study (Fig. S5). Additional studies on larger cohorts of patients are needed to specifically address this point.

**Table S1. Neuropathological profiles of AD cases**

| Case | Age  at onset | Age  at  death | ApoE/APP/PS1/PS2 genotype | Braak NFT stage | CAA | Plaque density score | Dispersion Index Frontal cortex | Aβ38 | Aβ40 | Aβ42 |
| --- | --- | --- | --- | --- | --- | --- | --- | --- | --- | --- |
| fAD1 | 36 | 46 | ε3/ε3, APP A673V | VI | 3 | 3 | 92,05 | ++ | ++ | ++ |
| fAD2 | 52 | 57 | ε3/ε3, APP A713T | VI | 4 | 2 | 123,3 | ++ | ++ | ++ |
| fAD3 | 36 | 43 | ε2/ε3, PS1 P117A | VI | 3 | 3 | 214,5 | - | + | +++ |
| fAD4 | 60 | 82 | ε3/ε3, PS2 A85V | VI | 0 | 3 | 239,3 | - | - | ++ |
| sAD1 | 79 | 82 | ε4/ε4 | V | 4 | 3 | 96,7 | + | ++ | + |
| sAD2 | 65 | 68 | ε3/ε3 | V-VI | 1-2 | 2 | 43,55 | +/- | ++ | ++ |
| sAD3 | n.a. | 59 | ε3/ε3 | VI | 1-2 | 3 | 143,76 | - | ++ | ++ |
| sAD4 | 79 | 81 | ε3/ε4 | III-IV | 2-3 | 3 | 108,53 | - | - | ++ |
| sAD5 | 77 | 83 | ε3/ε4 | VI | 3 | 1 | 153,84 | - | +/- | ++ |
| sAD6 | 60 | 75 | ε3/ε3 | VI | 1 | 3 | 65,21 | +/- | ++ | ++ |
| sAD7 | 62 | 72 | ε3/ε3 | VI | 3 | 3 | 240,3 | - | + | ++ |
| sAD8 | 62 | 68 | ε2/ε4 | III-IV | 2 | 2 | 78,38 | + | ++ | ++ |
| sAD9 | 83 | 86 | ε3/ε4 | IV | 3 | 3 | 218 | - | +/- | ++ |
| sAD10 | 85 | 90 | ε3/ε3 | III | 1 | 3 | 365,57 | - | +/- | ++ |
| sAD11 | 53 | 58 | ε2/ε3 | V-VI | 2 | 2 | 163,5 | - | + | ++ |
| sAD12 | 50 | 58 | ε3/ε3 | VI | 3 | 3 | 174,4 | - | +/- | ++ |
| sAD13 | 58 | 66 | ε4/ε4 | VI | 3 | 3 | 125,3 | +/- | + | ++ |
| sAD14 | 63 | 69 | ε2/ε4 | VI | 3 | 3 | 120 | - | +/- | ++ |
| sAD15 | 54 | 62 | ε2/ε3 | VI | 2 | 3 | 165,1 | - | +/- | ++ |
| sAD16 | 43 | 47 | ε4/ε4 | V-VI | 2-3 | 3 | 146,3 | - | + | ++ |
| sAD17 | 51 | 59 | ε3/ε4 | V | 2 | 3 | 133 | - | + | ++ |
| sAD18 | 47 | 61 | ε3/ε3 | VI | 3 | 3 | 157,3 | - | + | ++ |
| sAD19 | 69 | 72 | ε3/ε4 | V-VI | 2 | 3 | 277 | - | ++ | ++ |
| sAD20 | 82 | 86 | ε3/ε4 | IV | 3 | 2 | 118.9 | +/- | +/- | ++ |

**Figure S1**

**Fig. S1 Effects of immunodepletion of Aβ on aggregation kinetics of AD brain homogenates.** Soluble fractions from AD brain homogenates were diluted in 100 mM Tris-HCl pH 7.5, 5 μM ThT. ThT fluorescence is expressed as arbitrary units (a. u.).

**Figure S2**

**Fig. S2 RT-QuIC profiles of human brain extracts from the molecular subgroups of AD with Aβ1-40_WT_ as substrate.** Soluble fractions from AD brain homogenates were diluted in 100 mM Tris-HCl pH 7.5, 5 μM ThT, 10μM Aβ1-40. ThT intensity was normalized on the corresponding maximal ThT fluorescence and expressed as relative arbitrary units (a. u. %). Each brain was analyzed in quadruplicate; brains belonging to the same amyloid profile were grouped. Data are shown as mean ± SEM.

**Figure S3**

**

**

**Figure S4**

**
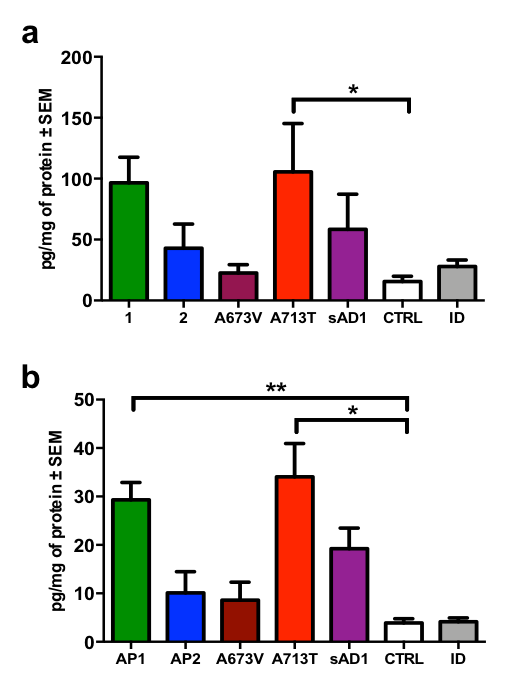
**

**Fig. S4 Aβ levels in insoluble fractions from brains of mice injected with human AD brain homogenates.** Aβ40 (a) and Aβ42 (b) were measured in duplicate by ELISA and expressed as pg per mg of total proteins. The results were compared by Kruskal-Wallis followed by Dunn’s multiple comparison test (*p<0.05; **p<0.01).

**Figure S5**

**
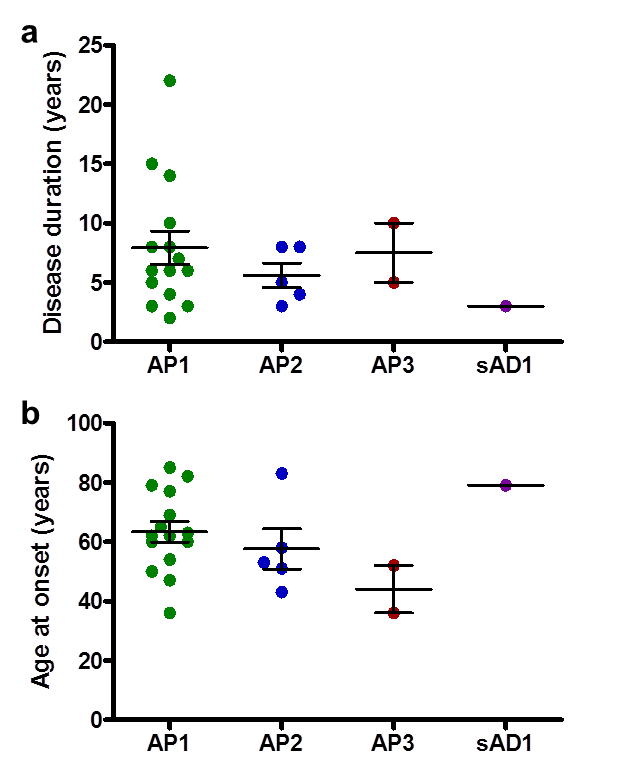
**

**Fig. S5 Correlation between AP profiles and disease duration (a) and age at onset (b).** The results were compared by Kruskal-Wallis followed by Dunn’s multiple comparison test.

**REFERENCES**

1 Di Fede, G. *et al.* A recessive mutation in the APP gene with dominant-negative effect on amyloidogenesis. *Science* **323**, 1473-1477, doi:10.1126/science.1168979 (2009).

2 Di Fede, G. *et al.* Good gene, bad gene: new APP variant may be both. *Prog Neurobiol* **99**, 281-292, doi:10.1016/j.pneurobio.2012.06.004 (2012).

3 Giaccone, G. *et al.* Neuropathology of the recessive A673V APP mutation: Alzheimer disease with distinctive features. *Acta Neuropathol* **120**, 803-812, doi:10.1007/s00401-010-0747-1 (2010).

4 Rossi, G. *et al.* A family with Alzheimer disease and strokes associated with A713T mutation of the APP gene. *Neurology* **63**, 910-912 (2004).

5 Kauwe, J. S., Wang, J., Chakraverty, S., Goate, A. M. & Henao-Martinez, A. F. Novel presenilin 1 variant (P117A) causing Alzheimer's disease in the fourth decade of life. *Neurosci Lett* **438**, 257-259, doi:10.1016/j.neulet.2008.04.029 (2008).

6 Piscopo, P. *et al.* A novel PSEN2 mutation associated with a peculiar phenotype. *Neurology* **70**, 1549-1554, doi:10.1212/01.wnl.0000310643.53587.87 (2008).

7 Hyman, B. T. *et al.* National Institute on Aging-Alzheimer's Association guidelines for the neuropathologic assessment of Alzheimer's disease. *Alzheimers Dement* **8**, 1-13, doi:10.1016/j.jalz.2011.10.007 (2012).

8 Montine, T. J. *et al.* Multisite assessment of NIA-AA guidelines for the neuropathologic evaluation of Alzheimer's disease. *Alzheimers Dement* **12**, 164-169, doi:10.1016/j.jalz.2015.07.492 (2016).

9 Alafuzoff, I. *et al.* Inter-laboratory comparison of neuropathological assessments of beta-amyloid protein: a study of the BrainNet Europe consortium. *Acta Neuropathol* **115**, 533-546, doi:10.1007/s00401-008-0358-2 (2008).

10 Heyman, A., Fillenbaum, G. G. & Mirra, S. S. Consortium to Establish a Registry for Alzheimer's Disease (CERAD): clinical, neuropsychological, and neuropathological components. *Aging (Milano)* **2**, 415-424 (1990).

11 Mirra, S. S. *et al.* The Consortium to Establish a Registry for Alzheimer's Disease (CERAD). Part II. Standardization of the neuropathologic assessment of Alzheimer's disease. *Neurology* **41**, 479-486 (1991).

12 Vonsattel, J. P. *et al.* Cerebral amyloid angiopathy without and with cerebral hemorrhages: a comparative histological study. *Ann Neurol* **30**, 637-649, doi:10.1002/ana.410300503 (1991).

13 Braak, H. & Braak, E. Neuropathological stageing of Alzheimer-related changes. *Acta Neuropathol* **82**, 239-259 (1991).

14 Braak, H., Alafuzoff, I., Arzberger, T., Kretzschmar, H. & Del Tredici, K. Staging of Alzheimer disease-associated neurofibrillary pathology using paraffin sections and immunocytochemistry. *Acta Neuropathol* **112**, 389-404, doi:10.1007/s00401-006-0127-z (2006).

15 Capetillo-Zarate, E. *et al.* Selective vulnerability of different types of commissural neurons for amyloid beta-protein-induced neurodegeneration in APP23 mice correlates with dendritic tree morphology. *Brain* **129**, 2992-3005, doi:10.1093/brain/awl176 (2006).

16 Garcia-Alloza, M. *et al.* Characterization of amyloid deposition in the APPswe/PS1dE9 mouse model of Alzheimer disease. *Neurobiol Dis* **24**, 516-524, doi:10.1016/j.nbd.2006.08.017 (2006).

17 Maderna, E. *et al.* Divergent cognitive status with the same Braak stage of neurofibrillary pathology: does the pattern of amyloid-β deposits make the difference? *J Alzheimers Dis* **43**, 375-379, doi:10.3233/JAD-140540 (2015).

18 DaRocha-Souto, B. *et al.* Brain oligomeric β-amyloid but not total amyloid plaque burden correlates with neuronal loss and astrocyte inflammatory response in amyloid precursor protein/tau transgenic mice. *J Neuropathol Exp Neurol* **70**, 360-376, doi:10.1097/NEN.0b013e318217a118 (2011).

19 Samaroo, H. D. *et al.* High throughput object-based image analysis of β-amyloid plaques in human and transgenic mouse brain. *J Neurosci Methods* **204**, 179-188, doi:10.1016/j.jneumeth.2011.10.003 (2012).
